# Polyphenols as Modulators of Oxidative Stress and Inflammation in Endometriosis: Molecular Mechanisms and Clinical Perspectives

**DOI:** 10.3390/antiox15091186

**Published:** 2026-09-17

**Authors:** Cristian Ioan Iuhas, Andreea Stănilă, Carmen Socaciu, Vlad N. Dudric, Corina Danciu, Melania Florina Munteanu, Răzvan Ciortea, Cristina Mihaela Ormindean, Andrei Măluțan, Zorița Diaconeasa, Dan Mihu, Liora Colobatiu

**Affiliations:** 1 2nd Department of Obstetrics and Gynecology, “Iuliu Hatieganu” University of Medicine and Pharmacy, 400012 Cluj-Napoca, Romania; iuhascristianioan@yahoo.co.uk (C.I.I.); r_ciortea@yahoo.com (R.C.); cristina.mihaela.prodan@gmail.com (C.M.O.); malutan.andrei@gmail.com (A.M.); dan.mihu@yahoo.com (D.M.); 2 Faculty of Food Science and Technology, University of Agricultural Sciences and Veterinary Medicine, 400372 Cluj-Napoca, Romania; stanila.andreea@usamvcluj.ro (A.S.); carmen.socaciu@usamvcluj.ro (C.S.); 3 Department of Surgery, “Iuliu Hatieganu” University of Medicine and Pharmacy, V-th Surgical Clinic, 400139 Cluj-Napoca, Romania; vlad.dudric@umfcluj.ro; 4 Department of Pharmacognosy, Faculty of Pharmacy, Victor Babeș University of Medicine and Pharmacy Timișoara, Eftimie Murgu Square, No. 2, 300041 Timișoara, Romania; corina.danciu@umft.ro; 5 Research and Processing Center of Medicinal and Aromatic Plants, Victor Babeș University of Medicine and Pharmacy Timișoara, Eftimie Murgu Square, No. 2, 300041 Timișoara, Romania; 6 Department of Pharmaceutical Sciences, “Vasile Goldiş” Western University of Arad, B-dul Revoluției 94-96, 310025 Arad, Romania; munteanu.melania@uvvg.ro; 7 Faculty of Pharmacy, “Iuliu Hatieganu” University of Medicine and Pharmacy, 400012 Cluj-Napoca, Romania; mihaela.colobatiu@umfcluj.ro

**Keywords:** endometriosis, polyphenols, oxidative stress, inflammation, resveratrol, quercetin

## Abstract

Endometriosis, a chronic inflammatory condition affecting up to 10% of reproductive-age individuals, is characterized by the presence of endometrial-like tissue outside the uterine cavity. Current treatments are based primarily on hormonal therapy, analgesia, and surgery, while additional biologically targeted approaches remain under investigation. This review examines polyphenols in relation to endometriosis-associated redox dysregulation and inflammation. Rather than acting simply as antioxidants, individual polyphenols may function as context-dependent redox and signaling modulators. Preclinical studies suggest effects on NF-κB and MAPK signaling, Nrf2-related stress responses, angiogenesis, apoptosis, immune-cell activity, and hormone-related pathways, but the direction and magnitude of these effects differ substantially among compounds, doses, metabolites, and experimental models. Resveratrol and quercetin have generated the greatest clinical interest; however, human evidence remains sparse and heterogeneous, quercetin has been evaluated mainly in multicomponent formulations, and the only double-blind placebo-controlled randomized trial of resveratrol did not demonstrate superiority over placebo for pain reduction. Low and variable bioavailability, compound-specific metabolism, phytoestrogenic and pro-oxidant effects at some exposures, heterogeneous formulations, and limited long-term safety data remain major translational barriers. Polyphenols should therefore be regarded as potential adjunctive approaches whose clinical efficacy in endometriosis remains to be established.

## 1. Introduction

Endometriosis is a chronic, benign, estrogen-dependent disorder marked by the presence of endometrial-like tissue outside the uterine cavity. These ectopic tissues, commonly found on the ovaries, fallopian tubes, and peritoneum, are capable of responding to hormonal changes during the menstrual cycle. This leads to cyclic bleeding, inflammation, fibrosis, and the formation of adhesions, which can cause debilitating symptoms [1,2].

Global epidemiological data indicates that endometriosis affects approximately 190 million individuals worldwide, with prevalence rates varying significantly across different populations and diagnostic criteria. Endometriosis represents a major public health concern [3]. Studies suggest that approximately 10% of reproductive-age women are affected, with this percentage rising to 35–50% among those experiencing pelvic pain and infertility. It is also becoming increasingly common among adolescents and among an unknown number of gender-diverse individuals [4]. While primarily affecting women during their reproductive years, the condition is often underdiagnosed or misdiagnosed due to its heterogeneous clinical presentation that often partially overlaps with other gynaecological or gastrointestinal conditions. Symptoms are highly variable and include pelvic pain ranging from mild to severe, fatigue, heavy menstrual bleeding, painful urination or bowel movements, and in many cases, infertility. Endometriosis is in fact one of the leading causes of female infertility [1].

In addition to physical discomfort and reduced quality of life, the disease has a marked impact on mental health and generates a considerable economic burden similar to that of chronic conditions like diabetes, with per-patient annual costs nearing €9600, driven by medical expenses and lost productivity. In the United States alone, this burden is estimated at $69.4 billion annually [3,5].

The exact etiology of the disease remains unclear; however, several theories have been proposed, among which Sampson’s theory of retrograde menstruation is the most widely accepted. The pathophysiology involves a complex interplay of hormonal, genetic, and immunological factors [2,5].

Oxidative stress in endometriosis is better understood as dysregulated redox homeostasis rather than simply an excess of oxidants that should be globally suppressed. Reactive oxygen species (ROS) are generated by erythrocyte/iron turnover, activated immune cells, mitochondria, and ectopic tissue within the peritoneal environment. At excessive or poorly controlled levels, ROS contribute to lipid peroxidation, protein oxidation, oxidative DNA lesions, and inflammatory signaling [6,7,8]. However, ROS also participate in physiological and stress-response signaling, and their biological consequences depend on concentration, cellular compartment, timing, and cell type. This distinction is particularly relevant in endometriosis because lesion cells and surrounding peritoneal cells may respond differently to the same redox pressure.

Chronic inflammation is closely linked to oxidative stress, as ROS act as secondary messengers that activate the NF-κB signaling pathway. This activation induces the production of pro-inflammatory cytokines (IL-1β, IL-6, IL-8, TNF-α) and chemokines (MCP-1). In addition, elevated levels of prostaglandin E2 (PGE2) are observed, which are associated with pain and further stimulate local estrogen production via the enzyme aromatase, resulting in a self-perpetuating cycle [5,6].

Current standard treatments for endometriosis are effective for many patients in reducing pain, suppressing disease activity, or restoring anatomy, but they have important limitations. Medical treatment is generally suppressive rather than curative, symptoms may recur after treatment discontinuation, and therapeutic choices must be individualized according to pain, fertility goals, adverse effects, lesion phenotype, and patient preference [3,5,9].

Hormonal therapies, including oral contraceptives, Gonadotropin-releasing hormone (GnRH) agonists, and progestins, aim to induce a hypoestrogenic state to inhibit lesion growth. However, these treatments do not eradicate the disease and instead provide only temporary symptom suppression, with pain frequently persisting or recurring after discontinuation. In addition, their contraceptive nature represents a significant limitation for patients seeking to conceive, as suppression of the ovarian function directly counteracts fertility goals. Moreover, long-term use is also associated with systemic adverse effects, including osteoporosis, vasomotor symptoms, alterations in lipid profiles, and potential liver dysfunction [3,5].

Surgical intervention, particularly laparoscopic excision of lesions, can provide substantial symptom relief and restore anatomy in selected patients. Nevertheless, recurrence remains clinically important, and symptoms may return after surgery or after cessation of adjunctive hormonal therapy [3]. Conventional management is therefore effective but not uniformly durable, and there is no established intervention that reliably prevents disease onset or progression. These limitations have encouraged investigation of additional mechanistically informed approaches that target inflammatory, angiogenic, hormonal, fibrotic, or redox pathways without implying that such approaches are established alternatives to standard care.

Polyphenols are naturally occurring plant-derived compounds with chemically and biologically diverse activities. Although they are often described collectively as antioxidants, this label is incomplete: individual polyphenols can influence redox-sensitive signaling, inflammatory pathways, proliferation, apoptosis, angiogenesis, and hormone-receptor activity, and some can display pro-oxidant behavior under particular concentrations or metal-rich conditions [10,11]. Their effects therefore depend on chemical structure, dose, metabolism, cellular context, and the surrounding redox environment.

Inflammation is a defining feature of endometriosis and contributes to pain, lesion survival, angiogenesis, fibrosis, and altered immune function. In this context, the ability of selected polyphenols to modulate inflammatory and redox-sensitive pathways has generated interest in their biological relevance to endometriosis; however, mechanistic activity should not be equated with demonstrated clinical benefit.

Polyphenols are of interest because they can modulate several intersecting biological processes implicated in endometriosis, including MAPK/ERK, PI3K/AKT, NF-κB, angiogenic, redox, and cell-survival pathways. This pleiotropic activity should not be interpreted as a simple contrast with conventional treatment, since hormonal therapies also exert multiple downstream effects and surgery acts through anatomical removal rather than a single molecular target. Preclinical studies suggest that several polyphenols can inhibit proliferation and migration, promote apoptosis, reduce VEGF-related angiogenesis, and modulate matrix metalloproteinases (MMPs). Among the most extensively studied compounds are curcumin, resveratrol, and epigallocatechin-3-gallate (EGCG); however, the strength of evidence differs substantially among compounds and is predominantly preclinical [9,12,13,14].

Despite increasing interest in polyphenols in endometriosis research, major barriers limit clinical translation. The compounds reviewed differ substantially in absorption, metabolism, circulating metabolites, tissue exposure, estrogen-receptor activity, and effective experimental concentrations. Current evidence is dominated by preclinical studies, often using exposures that are not directly comparable with achievable systemic concentrations, while human evidence remains sparse [10,12,13,15]. Standardized formulations, dose-ranging, pharmacokinetic characterization, long-term safety data, and compound-specific quality control are therefore required before clinical efficacy can be inferred.

The aim of this review is to critically evaluate evidence concerning individual polyphenols in endometriosis, with particular emphasis on redox signaling and inflammation. It examines compound-specific molecular mechanisms, distinguishes preclinical mechanistic evidence from the limited human literature, and discusses the pharmacokinetic, formulation, hormonal, safety, and methodological factors that currently constrain clinical translation.

Unlike earlier reviews focused primarily on dietary polyphenols or anti-inflammatory effects, the present review integrates oxidative-stress pathways, compound-specific molecular mechanisms, preclinical evidence, available human studies, formulation-related limitations, and translational priorities.

## 2. Materials and Methods

This paper is a structured narrative review of the mechanistic, preclinical, and human evidence concerning polyphenols in endometriosis, with particular emphasis on redox signaling and inflammation. The original literature search was conducted in Web of Science, PubMed, Scopus, and Google Scholar. The search used the following combinations: polyphenols AND endometriosis; polyphenols AND inflammation; polyphenols AND proinflammatory cytokines; polyphenols AND oxidative stress; polyphenols AND reactive oxygen species; polyphenols AND antioxidant enzymes; quercetin AND endometriosis; resveratrol AND endometriosis; polyphenols AND endometriosis AND in vitro; and polyphenols AND endometriosis AND in vivo. No publication-date or article-type filters were applied, and the search was last updated in May 2026.

The records identified by the original search were screened on the basis of title, abstract, and full-text availability. Non-English publications were excluded. Screening was performed independently by two authors, and disagreements were resolved by discussion with a third author. These procedures reflect the search and screening information documented in the original manuscript.

The review was not prospectively designed or registered as a systematic review, and exact record-level counts for identification, deduplication, exclusion, and final inclusion were not available. Therefore, numerical PRISMA counts and a study-selection flow diagram were not reconstructed, in order to avoid introducing potentially inaccurate information. The evidence is consequently presented as a structured qualitative summary of the identified and verified preclinical literature.

## 3. Endometriosis: Pathophysiology and Role of Inflammation

Endometriosis is a chronic gynecologic condition affecting up to 10% of women of reproductive age. Genome-wide association studies have identified 10 genomic regions linked to endometriosis. These studies account for less than 4% of heritability, with approximately half of the heritability attributed to common risk variants [16]. The main and most reported symptoms by patients with endometriosis include pain (pelvic pain), fatigue, menorrhagia, dysmenorrhea (painful periods), dysuria, gastrointestinal distress, dyspareunia (painful intercourse) and infertility. Alongside physical discomfort, most patients also face prevalent socio-economic challenges, particularly in low- and middle-income countries. The high costs of diagnosis and treatment, combined with the overlap between endometriosis symptoms and menstrual symptoms, hinder early diagnosis of the disease. Additionally, for trans and gender-diverse individuals, there are further barriers such as poorer access to gender-affirming healthcare, higher rates of mental health concerns and discrimination in areas like housing, employment and social support—serious social factors that obstruct early detection of the condition [4,17].

From a medical perspective, endometriosis is characterized by the presence of pseudoglandular endometrial cells (endometrial-like tissue) outside the uterine cavity. The infiltration of these cells and tissues into the extrauterine space causes peritoneal lesions, superficial implants, nodules or cysts at the level of the ovary, or even deep infiltrating disease. They are located at the level of pelvic and even abdominal peritoneum, in deep pelvic structures like vessels, nerves, bowels, bladder wall, and sometimes even distant organs with subsequent distortion of the local anatomy resulting from adhesion formation, a repair process that impairs the function and the sensitivity of the affected areas [1,17].

From a biological perspective, endometriosis arises through a complex interplay of genetic, hormonal, immunological, and environmental factors. Retrograde menstruation can introduce endometrial cells, erythrocytes, haemoglobin, and iron into the peritoneal cavity. Iron-catalysed redox reactions can increase ROS production and promote lipid peroxidation, protein oxidation, and oxidative DNA lesions [5,7,8]. Oxidative DNA damage can contribute to genomic instability and mutagenic pressure, but it should not be equated directly with the acquisition of established somatic mutations. Recurrent somatic alterations identified in endometriotic lesions reflect clonal processes whose origins and selection are multifactorial. Thus, the iron-rich inflammatory microenvironment provides a plausible source of oxidative genomic stress rather than a demonstrated linear sequence in which DNA degradation in peritoneal fluid directly causes lesion-specific somatic mutations.

Some theories suggest that immune-system dysregulation may contribute not only to progression but also to the establishment of endometriosis, creating a self-reinforcing inflammatory environment. Activated macrophages accumulate in the peritoneal cavity and would normally participate in clearance of refluxed endometrial cells; however, reduced phagocytic activity has been reported in women with endometriosis. At the same time, increased production of pro-inflammatory cytokines and increased COX-2 expression promote prostaglandin synthesis, inflammation, nociception, and local estrogenic signaling, thereby facilitating survival and growth of endometriotic cells [1,5].

As discussed earlier, macrophages play a crucial role in the development of endometriotic pain by releasing pain-inducing molecules, such as prostaglandins, which are involved in maintaining neuropathic pain associated with endometriosis. Alongside the increased presence of macrophages in the peritoneal cavity, elevated levels of COX-1 and COX-2 enzymes have been observed in ectopic endometrial lesions in rodent models. Upregulated COX-2 facilitates the production of the inflammatory mediator prostaglandin E2 (PGE2), a key player in the pathophysiology of endometriosis [5,18].

The temporal role of immune dysfunction in endometriosis remains unresolved. Altered macrophage function, reduced natural-killer-cell cytotoxicity, changes in T-cell responses, cytokine production, and pro-angiogenic signaling have all been described, but it remains uncertain which abnormalities contribute to lesion establishment, which develop secondarily to persistent ectopic tissue, and which primarily promote progression of established disease [5]. Neutrophils and macrophages can produce mediators that support angiogenesis and lesion growth, while reduced clearance of refluxed endometrial cells has also been proposed. These observations support immune dysregulation as an important component of the disease network rather than a single demonstrated initiating event.

Accordingly, the pathogenesis of endometriosis should not be represented as a linear sequence from retrograde menstruation to oxidative stress, mutation, immune escape, and lesion formation. Retrograde menstruation, iron accumulation and redox stress, estrogen-dependent signaling, immune-cell dysfunction, angiogenesis, fibrosis, neurogenesis, and lesion-specific genetic or epigenetic alterations are interacting processes whose temporal and causal relationships remain incompletely resolved [5,7,16,19]. Inflammation and MAPK signaling are important components of this network, but their contribution varies across disease stages, lesion phenotypes, and cellular compartments.

### The Redox Microenvironment of Endometriotic Lesions

Although chronic inflammation is common to many benign proliferative disorders, the redox environment of the endometriotic pelvis has features that distinguish it from generic inflammation and are directly relevant to redox-directed interventions. Retrograde menstruation and repeated cyclic bleeding within established lesions deliver erythrocytes into the peritoneal cavity, where haemolysis releases haemoglobin and subsequently free haem and catalytically active iron. Iron concentrations in peritoneal fluid, ovarian endometriomas, and lesion-resident macrophages are consistently elevated relative to controls [7].

Free ferrous iron drives Fenton and Haber–Weiss chemistry, converting hydrogen peroxide into the highly reactive hydroxyl radical. Because the peritoneal fluid is an aqueous, electrolyte-rich compartment with limited buffering capacity, the resulting radical flux acts on membrane phospholipids, proteins and nuclear DNA of both ectopic cells and infiltrating immune cells. Lipid peroxidation generates reactive aldehydes such as malondialdehyde (MDA) and 4-hydroxynonenal (4-HNE), which form protein and DNA adducts, amplify NF-κB signalling and are detectable at increased concentrations in the peritoneal fluid of affected women [6,8].

A second, more recently characterised feature is the relationship between this iron-rich environment and ferroptosis, an iron-dependent form of regulated cell death driven by lipid hydroperoxide accumulation and restrained by glutathione peroxidase 4 (GPX4) and the cystine-glutamate antiporter system x^c−^. Experimental and observational literature suggests that endometriotic stromal cells may acquire relative resistance to ferroptotic stress despite persistent iron excess, while neighbouring peritoneal cells can remain vulnerable [7,8]. This observation raises an emerging mechanistic hypothesis: selectively increasing ferroptotic vulnerability in lesion cells while limiting oxidative injury to surrounding tissue could become therapeutically relevant. It should not, however, be regarded as an established treatment principle because selective ferroptosis restoration has not been demonstrated clinically in endometriosis. The same uncertainty also cautions against assuming that indiscriminate antioxidant supplementation is uniformly beneficial, an issue considered further in Section 6.

Systemic and local antioxidant-defense systems are also altered. Reduced activity of superoxide dismutase, catalase and glutathione peroxidase, together with lower total antioxidant capacity, has been reported in the serum and peritoneal fluid of women with endometriosis [6]. These observations indicate disturbed redox homeostasis, but they do not establish that maximal ROS suppression is therapeutically desirable. In an iron-rich lesion microenvironment, ROS can contribute simultaneously to tissue injury, inflammatory signaling, and regulated cell-death pathways such as ferroptosis. The relevant therapeutic question is therefore whether a compound can beneficially reshape cell-specific redox signaling rather than whether it is simply capable of scavenging radicals. This conceptual distinction provides the rationale for the Nrf2/KEAP1 and direct-versus-indirect antioxidant mechanisms discussed in Section 4.2.

## 4. Overview of Polyphenols

Polyphenols are an umbrella chemical category rather than a single pharmacological class. Their biological activities can include redox modulation, anti-inflammatory signaling, effects on proliferation and metabolism, and interactions with hormone receptors, but these properties are not shared uniformly across all compounds [11,20]. Structural differences determine absorption, conjugation, microbial metabolism, receptor affinity, cell permeability, and achievable tissue exposure. Accordingly, references in this review to recurring ‘polyphenol effects’ describe mechanistic themes observed across selected compounds and should not be interpreted as evidence of a uniform class effect.

### 4.1. Classification, Chemical Structure and Sources

Polyphenols are a large and chemically diverse group of plant-derived compounds that are commonly classified into flavonoids, phenolic acids, lignans, and stilbenes (Table 1). These structures contain one or more phenolic rings bearing hydroxyl groups and other substituents whose number and position influence redox behaviour, solubility, metabolism, and molecular interactions. Additional phenolic compounds fall outside these four broad classes because of distinct structural features [11,21].

### 4.2. Molecular Mechanisms Underlying the Anti-Inflammatory Effects of Polyphenols in Endometriosis

Polyphenols exert anti-inflammatory effects through the modulation of multiple molecular pathways involved in both oxidative stress and chronic inflammation. In endometriosis, persistent activation of inflammatory signaling contributes to ectopic lesion survival, angiogenesis, fibrosis, immune dysregulation, and pain. Consequently, the therapeutic relevance of polyphenols extends beyond their general anti-inflammatory activity, as they target several pathways directly implicated in endometriosis pathogenesis, including NF-κB, MAPK, COX-2/PGE2, and Nrf2 signaling. The principal mechanisms through which polyphenols may interfere with endometriosis-associated inflammation are discussed below.

#### 4.2.1. Inhibition of NF-κB Activation

The nuclear factor kappa B (NF-κB) pathway is a key transcriptional regulator of inflammatory responses and controls genes encoding mediators such as interleukin-6 (IL-6), tumor necrosis factor-alpha (TNF-α), and interleukin-1 beta (IL-1β). Polyphenols including curcumin and resveratrol have been reported to reduce NF-κB signaling in experimental systems, including through effects on IκBα phosphorylation/degradation and related upstream signaling [20,26].

Since NF-κB is constitutively activated in endometriotic lesions and contributes to the production of IL-1β, IL-6, IL-8, TNF-α, MCP-1, and other inflammatory mediators, its inhibition represents one of the principal mechanisms through which polyphenols may reduce lesion growth and inflammation.

#### 4.2.2. Inhibition of Pro-Inflammatory Enzymes

Several polyphenols reduce the COX-2/PGE2 and iNOS/NO inflammatory axes, but decreased enzyme expression should be distinguished from direct inhibition of catalytic activity. In endometriosis-related experimental models, apigenin has been reported to reduce TNF-α-induced COX-2 expression and PGE2 production, whereas puerarin reduces COX-2/PGE2 signaling in vivo [9,18,27]. Accordingly, the available evidence more consistently supports downregulation of expression or pathway output than a universal direct enzymatic inhibitory effect. This distinction is relevant because COX-2-derived PGE2 contributes both to nociception and to local estrogen biosynthesis through aromatase-related signaling.

#### 4.2.3. Suppression of MAPK Pathways

Mitogen-activated protein kinases (MAPKs), including ERK, JNK, and p38, play pivotal roles in inflammatory signaling, proliferation, migration, and stress responses. MAPK cascades can be activated by oxidative stress and by cytokine-receptor signaling. Mechanistically, IL-1β binds the IL-1 receptor and signals through a TIR-domain/MyD88-associated pathway, whereas TNF-α signals through members of the TNF-receptor superfamily; neither pathway is accurately described as signaling through a classical receptor tyrosine kinase. These receptor systems converge on downstream MAPK and NF-κB pathways and can increase inflammatory cytokines and PTGS2/COX-2 expression [28]. EGCG has been reported to suppress MAPK signaling in endometriotic stromal cells, with associated reductions in proliferation, migration, invasion, and fibrogenic responses [29].

#### 4.2.4. Modulation of Nrf2 Signaling

The nuclear factor erythroid 2-related factor 2/Kelch-like ECH-associated protein 1 (Nrf2/KEAP1) pathway is a major regulator of cellular responses to electrophilic and oxidative stress and controls genes including NQO1 and HO-1, together with broader antioxidant and detoxification programs [6]. Re-examination of the original naringenin study by Kapoor et al. showed that, in a rat model of endometriosis, naringenin increased Nrf2 and the downstream proteins NQO1 and HO-1 while decreasing the repressor KEAP1; these changes occurred together with reduced lesion burden and induction of apoptosis [30]. This direction of effect corrects the previous description in Table 2. Importantly, Nrf2 modulation in endometriosis should be interpreted in a cell- and context-dependent manner: reinforcement of antioxidant defenses may protect the surrounding peritoneal environment, whereas persistent antioxidant adaptation within lesion cells could theoretically facilitate survival under chronic iron/ROS stress. Therefore, Nrf2 activation is not in itself synonymous with a uniformly beneficial anti-endometriotic effect.

#### 4.2.5. Direct Radical Scavenging Versus Indirect Antioxidant Action

A mechanistic distinction that is frequently blurred in the literature is that between direct radical scavenging and indirect redox signaling. In cell-free chemical assays, many polyphenols can donate hydrogen atoms or electrons and directly quench reactive species. In vivo, however, parent aglycones are extensively metabolized and generally reach concentrations far below those used in many in vitro experiments, while endogenous antioxidants such as urate, ascorbate, and glutathione are present at substantially higher concentrations [10]. Direct radical scavenging by the parent compound is therefore unlikely to explain all biological effects observed after oral exposure.

An indirect mechanism is biologically plausible for some polyphenols and metabolites. Electrophilic or redox-active species can modify KEAP1 cysteine residues and thereby favour Nrf2 nuclear translocation and transcription of cytoprotective genes. However, the magnitude, concentration range, persistence, and even direction of this response are compound-, metabolite-, cell-, and disease-context-dependent and should not be generalized to the entire polyphenol class. The naringenin findings discussed above illustrate this complexity: increased Nrf2/NQO1/HO-1 signaling coexisted with ROS-associated mitochondrial apoptosis and reduced lesion burden [30].

This distinction also bears on which chemical species should be studied. Following absorption, polyphenols are extensively conjugated in the enterocyte and liver to glucuronides, sulfates and methylated derivatives, while the substantial unabsorbed fraction reaching the colon is catabolised by the gut microbiota into smaller phenolic acids and, in the case of ellagitannins and isoflavones, into urolithins and equol. These circulating metabolites differ from the parent compounds in redox potential, receptor affinity and cell permeability, and inter-individual variation in microbiota composition produces correspondingly variable metabolite profiles [10]. The near-universal practice of exposing endometriotic cells to parent aglycones in vitro is therefore a plausible contributor to the preclinical-to-clinical translation gap discussed in Section 7, and future in vitro work should include physiologically achievable concentrations of the relevant conjugated and microbial metabolites.

## 5. Polyphenols and Endometriosis: Current Evidence

Polyphenols comprise a chemically heterogeneous group, and evidence should therefore be interpreted compound by compound rather than as a class-wide therapeutic signal. Resveratrol, quercetin, curcumin, EGCG, genistein, naringenin, puerarin, and other compounds differ in bioavailability, metabolism, receptor binding, redox behavior, and experimental exposure ranges. Of particular relevance to an estrogen-dependent disease, resveratrol, genistein, and related structures may display estrogen-receptor agonistic or antagonistic effects depending on dose, receptor subtype, and hormonal context [20,31]. Table 2 therefore presents a structured qualitative summary of preclinical evidence for individual compounds only, separated into in vitro and animal evidence. Human studies are shown separately in Table 3 so that clinical design and risk-of-bias considerations are not conflated with molecular or animal outcomes.

**Table 2:** Preclinical evidence for individual polyphenols in endometriosis: in vitro and animal studies.

Compound	Experimental Model	Reported Molecular/Biochemical Changes	Reported Phenotypic Outcomes	References
PRECLINICAL EVIDENCE—IN VITRO MODELS				
Apigenin	- HPV16 E6/E7-immortalized normal vaginal (VK2/E6E7) and endocervical (End1/E6E7) epithelial surrogate lines; not ectopic-lesion-derived - Endometriotic stromal cells - Primary endometriotic stromal cells from ovarian endometrioma	↓COX-2 ↓PGE2 ↓IL-8 ↓NF-κB ↓ERK1/2 and JNK ↓Mitochondrial membrane potential ↑Calcium ions in cytosol ↑BAX ↑Cyt-c ↑ROS and ER stress	↓Cell proliferation ↓Inflammation ↑Apoptosis	[18,32]
Baicalein	- Endometrial stromal cells from patients with ovarian endometriosis - Ectopic endometrial stromal cells	↓BCL-2 ↓NF-κB ↓Cells in the S and G2/M phases ↓MMP-9, MMP-2, MT1-MMP ↓TGFB1 ↓FURIN ↑Cells in the G1 phase	Cell cycle arrest ↓Cell viability ↓Cell invasiveness ↑Apoptosis	[33,34]
Curcumin	- Primary endometriotic stromal cells - Primary endometriotic stromal cells derived from eutopic endometrium - Endometriotic stromal cells	↓ICAM1 ↓VCAM1 ↓IL-6, IL-8 ↓MCP-1 ↓NF-κB ↓E2 production ↓Cells in the S phase ↓VEGF ↓IP-10 ↓G-CSF ↓RANTES ↑Cells in the G1 phase	Cell cycle arrest ↓Inflammation ↓Cell proliferation ↓Angiogenesis ↑Apoptosis ↑Cell adhesion	[35,36,37,38]
EGCG	- Endometriotic and endometrial stromal cells from patients	↓MAPK signaling ↓Smad signaling ↓αSMA ↓Col-I ↓CTGF ↓FN	↓Cell proliferation ↓Cell migration ↓Cell invasion ↓Fibrogenesis	[29]
Naringenin	- HPV16 E6/E7-immortalized normal vaginal (VK2/E6E7) and endocervical (End1/E6E7) epithelial surrogate lines; not ectopic-lesion-derived [39]	↓Mitochondrial membrane potential ↓ROS ↓MMP-2	↓Cell proliferation ↑Apoptosis	[19]
Puerarin	- Primary endometriotic stromal cells - Chick chorioallantoic membrane model - Endometriotic stromal cells	↓MMP-9 ↓ICAM-1 ↓VEGF ↓CCND1/Cyclin D1 ↓CDC25A ↓ERα coactivators (SRC-1, SRC-3) ↑ERα corepressors (SMRT, NCoR) ↑TIMP-1	↓Cell invasion ↓Angiogenesis ↓Cell proliferation ↑Antiestrogen activity	[40,41]
Quercetin	- HPV16 E6/E7-immortalized normal vaginal (VK2/E6E7) and endocervical (End1/E6E7) epithelial surrogate lines; not ectopic-lesion-derived [39]	DNA fragmentation ↓Mitochondrial membrane potential ↓ERK1/2 ↓p38 ↓AKT	↓Cell proliferation ↑Apoptosis	[42]
Resveratrol	- Primary endometriotic stromal cells - Primary endometriotic epithelial cells - Endometrial Ishikawa cell line - Primary eutopic and ectopic endometrial stromal cells from endometriosis patients - 3D primary endometriotic and endometrial cell cultures	Dose-dependent agonistic and antagonistic activity DNA fragmentation ↓Cell invasion in Matrigel ↓Cell number ↓IGF-1 ↓HGF ↓NO ↓MCP-1 ↓IL-6, IL-8 ↓RANTES ↑P53 ↑BAX ↑CASP3 ↑SIRT1	Estrogenic activity ↓Cell invasion ↓Cell proliferation ↓Angiogenesis ↓Inflammation ↑Apoptosis	[43,44,45,46,47,48]
Wogonin	- hTERT-immortalized human endometrial stromal line T-HESC; endometrial stromal origin, not ectopic-lesion-derived [49] - Primary stromal cultures from endometrial biopsies of women with endometriosis [31]	↓ERα ↑Cells in the G2/M phase	↓Cell proliferation ↑G2/M cell-cycle arrest	[31]
PRECLINICAL EVIDENCE—ANIMAL MODELS				
Apigenin	- Murine endometriosis model	↓Peritoneal VEGF ↓Peritoneal TNF-α ↓Peritoneal IL-6	↓Angiogenesis ↓Endometriotic implant volume ↓Inflammation	[50]
Baicalein	- Murine endometriosis model	↓MT1-MMP ↓FURIN ↓TGFB1	↓Cell invasiveness ↓Endometriotic lesion growth	[33]
Curcumin	- Murine endometriosis model	Cytochrome c-mediated mitochondrial pathway modulation p53-dependent and -independent pathway modulation ↓NF-κB ↓MMP-3, MMP-9 ↓TNF-α ↓Lipid and protein oxidation ↓VEGF ↓Microvessel density	↓Endometriotic lesion growth ↓Inflammation ↓Oxidation ↓Endometriotic tissue weight and volume ↓Angiogenesis ↑Apoptosis	[51,52,53]
EGCG	- Murine endometriosis model	↓Microvessel number and size ↓VEGFC/VEGFR2 expression and signalling ↓Lesion size and weight ↓Serum VEGF ↓α-SMA ↓CD31 ↑Apoptotic cells in lesions	↓Endometriotic implant/lesion size and growth ↓Angiogenesis ↑Apoptosis	[54,55]
Fisetin	- Murine endometriosis model	↓Inflammasome pathway ↓NF-κB ↓TNF-α ↓Mast cell activation ↓α-SMA ↓TGF-β ↓MDA ↓ROS,	↓Endometriotic lesion growth ↓Fibrotic process ↓Oxidation ↓Inflammation ↑Apoptosis	[56]
Genistein	- Murine endometriosis model	Antagonistic estrogen activity	↓Endometriotic implant size	[57]
Naringenin	- Murine endometriosis model	↓MMP-2, MMP-9 ↓TNF-α ↓Mitochondrial membrane potential ↓VEGF ↓BCL-2 ↓PCNA ↑Nrf2 ↑NQO1 ↑HO-1 ↓KEAP1 ↓TAK1 ↓PAK1 ROS-mediated apoptosis	↓Endometriotic lesion growth ↓Angiogenesis ↓Inflammatory signaling ↓Proliferation/invasion ↑Apoptosis	[30]
Oleuropein	- Murine endometriosis model	↓ERβ	↓Cell proliferation ↓Endometriotic lesion growth ↑Apoptosis	[58]
Puerarin	- Murine endometriosis model	↓CYP19A1 ↓Aromatase activity ↓HSD17B1 ↓COX-2 ↓PGE2 ↓ERβ ↓E2 ↑HSD17B2	↑Antiestrogen activity	[27]
Quercetin	- Murine endometriosis model	↓CCND1 ↓Cyclin D1 ↓Serum E2 ↓Serum FSH and LH levels ↓Serum oestrogen content ↓Serum TNF-α ↓ERα, ERβ ↓ERK1/2, P38 MAPK and AKT ↓mTOR ↑NQO1 enzyme activity ↑NRF2 ↑BECN1 ↑ATG5	↓Cell proliferation ↓Endometriotic lesion size ↓Oxidative stress ↓Angiogenesis ↓Inflammation ↑Autophagy ↑Cell cycle arrest	[42,59]
Resveratrol	- Murine endometriosis model - Immunocompromised mouse endometriosis model (RAG-2 knockout)	DNA fragmentation, EMT process suppression ↓PCNA ↓CD34 ↓Peritoneal VEGF ↓Vascular density area ↓MKI67 ↓ERα ↓Ki-67 ↓MTA1 ↓ZEB2 ↓MCP-1 in peritoneal fluid and plasma ↓VEGF in peritoneal fluid and endometriotic tissue ↑SOD activity in serum and tissue ↑GPX activity in serum and tissue	↓Endometriotic lesion number, size and volume ↓Cell proliferation ↓Cell migration ↓Cell invasion ↓Inflammation ↓Oxidation ↓Angiogenesis ↑Apoptosis	[43,47,60]
Wogonin	- Murine endometriosis model	↓Endometriotic implant size ↓Cell proliferation ↑Apoptosis	↓Proliferating cells ↑Apoptotic cells	[31]
Xanthohumol	- Murine endometriosis model	↓PI3K ↓Microvessel density ↓MAPK	↓Endometriotic lesion growth ↓Angiogenesis	[61]

Interpretive note for Table 2: this table contains preclinical evidence only. Molecular/biochemical changes are separated from phenotypic outcomes, and in vitro evidence is separated from animal evidence. Rows may aggregate more than one experiment for a compound; concentrations, treatment duration, route of administration, and model characteristics vary substantially and should not be interpreted as dose-equivalent or quantitatively comparable. Human evidence is presented separately in Table 3.

Across the preclinical literature, many studies report partially convergent effects, including reduced inflammatory signaling, inhibition of VEGF-related angiogenesis, modulation of MAPK/PI3K/AKT pathways, reduced proliferation, and increased apoptosis. These findings should nevertheless be interpreted cautiously because the evidence is heterogeneous in compound identity, experimental model, exposure concentration, treatment duration, route of administration, and measured endpoint. Convergence at the level of broad biological categories does not imply dose equivalence, model equivalence, or uniform efficacy across polyphenols.

Model heterogeneity is an additional limitation. The evidence base includes patient-derived ectopic endometriotic stromal or epithelial cells, eutopic endometrial cells from patients with endometriosis, immortalized epithelial or stromal surrogate lines, three-dimensional tissue systems, and surgically induced animal models. These systems are not interchangeable. In particular, immortalized vaginal/endocervical epithelial models such as VK2/E6E7 and End1/E6E7 cannot reproduce the mixed stromal, epithelial, vascular, and immune microenvironment of an ectopic human lesion, and surgically induced rodent lesions do not fully reproduce spontaneous human disease. Exposure differences further limit direct cross-study comparison.

### 5.1. Specific Polyphenols of Interest in Endometriosis

Based on the available evidence, resveratrol and quercetin are among the most extensively investigated polyphenols for the treatment of endometriosis. Accordingly, this section focuses on these two compounds. To the best of our knowledge, they are currently the only polyphenols that have been evaluated in clinical studies involving patients with endometriosis.

#### 5.1.1. Quercetin

**Figure 1:**
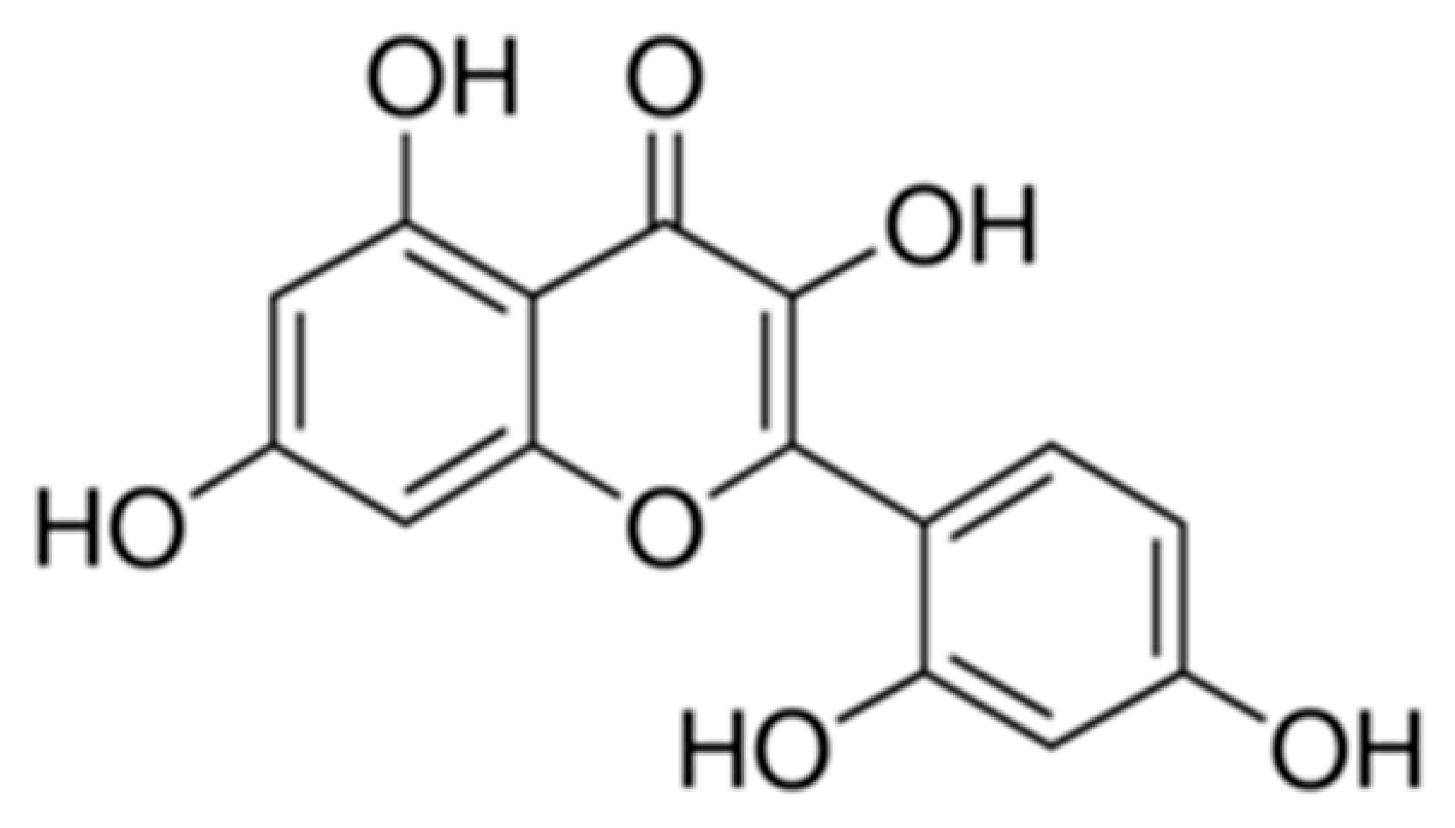
Quercetin (3,3′,4′,5,7-pentahydroxyflavone) (MW 302.24 g/mol).

Quercetin (3,3′,4′,5,7-pentahydroxyflavone, Figure 1) is ubiquitously present in fruits and vegetables and is therefore one of the most common dietary flavonols in the Western diet. After quercetin ingestion through vegetables and/or fruits, quercetin glycosides are metabolized, absorbed, and circulated in the blood. Once in the bloodstream, quercetin undergoes conjugation processes and is transported to the liver, where it is further modified. After these modifications, quercetin re-enters the circulation and is distributed to various tissues, including the brain and muscles, where it can exert its physiological effects [10,11].

In vitro and in vivo studies suggest that quercetin can influence PI3K/AKT-, MAPK/ERK-, cell-cycle-, autophagy-, and hormone-related pathways. Importantly, some frequently cited in vitro studies used VK2/E6E7 and End1/E6E7 cells, which are HPV16 E6/E7-immortalized epithelial lines derived from normal vaginal and endocervical epithelium rather than ectopic endometriotic lesions [39,42]. Findings from these surrogate models therefore require confirmation in patient-derived ectopic cells. In murine models, quercetin has been associated with reduced lesion size, inflammatory mediators, and angiogenic signaling, together with changes in estrogen-receptor expression and autophagy-related markers [42,59]. Outside endometriosis-specific models, quercetin has also been reported to attenuate mast-cell activation downstream of FcεRI and other activating receptors [62]; this general immunological evidence should not be interpreted as direct demonstration of mast-cell modulation within human endometriotic lesions.

Available human evidence for quercetin is derived from multicomponent formulations rather than quercetin monotherapy. Consequently, reductions in pain scores, PGE2, or CA-125 cannot be attributed independently to quercetin. PGE2 remains mechanistically relevant because it contributes to local estrogen biosynthesis, cell proliferation, inflammatory signaling, nociception, and angiogenesis [5,63].

PGE2 enhances estrogen production by increasing the expression of steroidogenic acute regulatory protein (StAR) and aromatase. It suppresses apoptosis and increases the levels of fibroblast growth factor-9 (FGF-9), thereby promoting cell proliferation. PGE2 also influences leukocyte populations and stimulates angiogenesis by modulating estrogen levels and upregulating vascular endothelial growth factor (VEGF) [63]. CA-125, also known as mucin 16 (MUC16), is a glycoprotein encoded by the MUC16 mucin gene. It is expressed by epithelial ovarian tumors and other pathological and normal tissues of Müllerian origin and can be detected in the serum. It may support the clinical assessment and monitoring of endometriosis, although it lacks sufficient sensitivity and specificity for use as a standalone diagnostic marker [64].

The controlled study by Signorile et al. included 90 women with stage IV endometriosis allocated to three groups of 30: a quercetin-containing multicomponent nutraceutical, a partial-formulation comparator, or placebo for 3 months [14]. The intervention combined quercetin with omega-3/omega-6 fatty acids, nicotinamide, 5-methyltetrahydrofolate, turmeric, and parthenium; improvements in symptoms and reductions in PGE2 and CA-125 were reported, but the contribution of quercetin cannot be isolated and the report provides limited detail on allocation concealment and blinding. Fadin et al. evaluated 33 women receiving quercetin 200 mg/day together with turmeric extract and N-acetylcysteine for 2 months and reported reductions in dysmenorrhea, pelvic pain, dyspareunia, and NSAID use [65]. This study was open-label and uncontrolled. These findings are therefore hypothesis-generating and do not establish efficacy of quercetin monotherapy.

#### 5.1.2. Resveratrol

**Figure 2:**
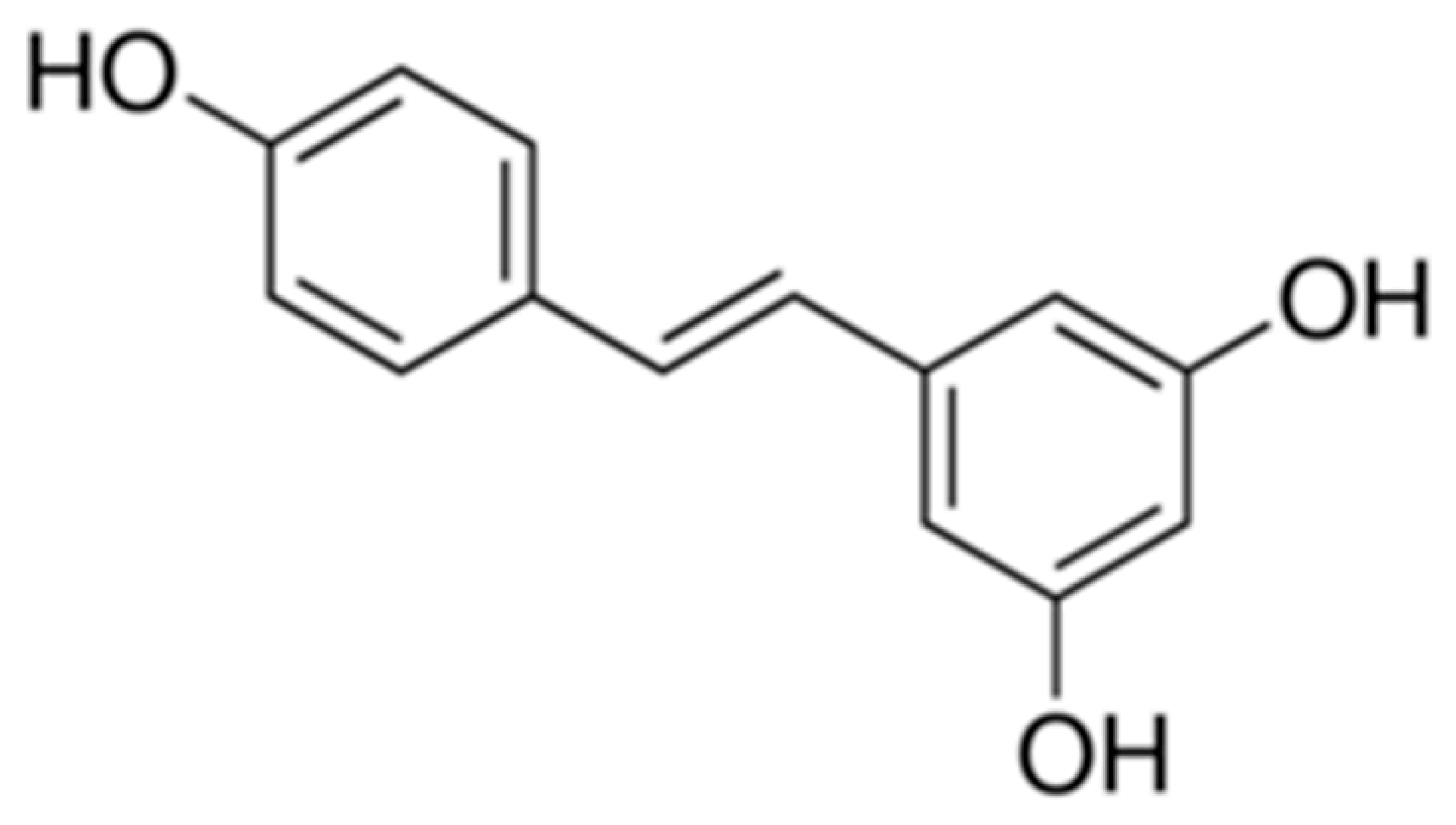
Resveratrol (3,4′,5-trans-trihydroxystilbene) (MW 228.24 g/mol).

Resveratrol (3,4′,5-trans-trihydroxystilbene, Figure 2) is a naturally occurring stilbene found in more than 70 plant species and plant-derived products, including grapes, berries, peanuts, and red wine. It acts as both a phytoalexin and a phytoestrogen [20,25].

Resveratrol has been investigated in patient-derived stromal cells, immortalized endometrial/endometriotic models, three-dimensional cultures, and experimental animal models. Primary studies report reduced stromal-cell invasiveness, inflammatory cytokine expression, proliferation, angiogenesis, and lesion growth, with effects on SIRT1-related signaling, apoptosis, extracellular-matrix remodeling, and EMT-associated pathways [43,44,45,46,47,48,60]. The magnitude and direction of these effects depend strongly on model and exposure; for example, in vitro concentrations are commonly in the micromolar range, whereas animal studies use distinct systemic dosing regimens. These preclinical findings therefore support mechanistic plausibility but should not be directly translated into a clinical dose or expected therapeutic effect.

**Figure 3:**
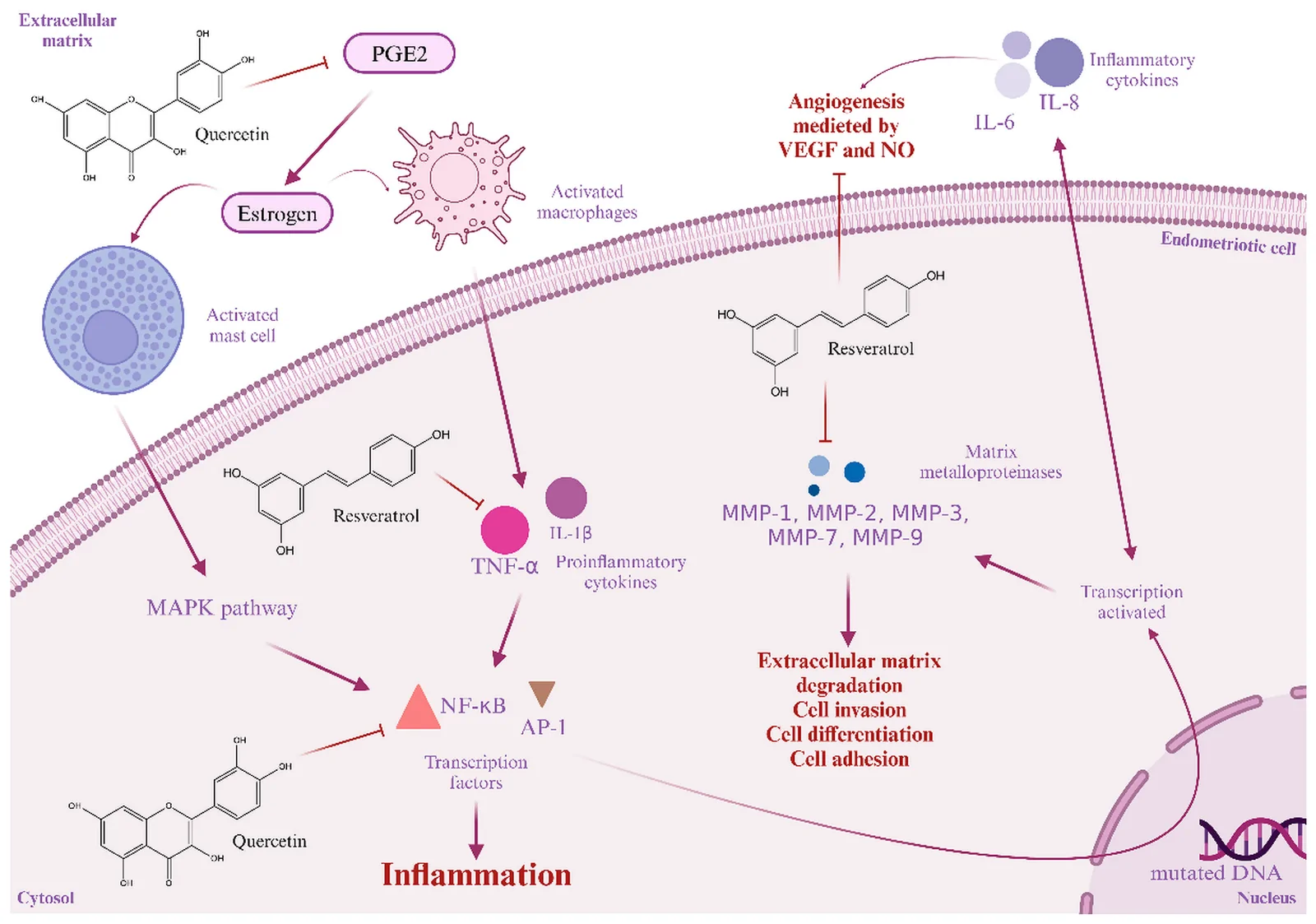
Schematic representation of molecular pathways reported in studies of quercetin and resveratrol relevant to endometriosis. Arrows summarize associations reported predominantly in in vitro and animal studies; selected human studies assessed only a limited subset of molecular endpoints and do not validate the entire pathway network clinically. The redox/Nrf2/ROS axis is discussed separately in The Redox Microenvironment of Endometriotic Lesions and Section 4.2.4 and Section 4.2.5 because its direction and biological significance are cell- and context-dependent and cannot be represented accurately as a single unidirectional effect. Original figure prepared by the authors.

Human studies of resveratrol provide a substantially weaker and more heterogeneous evidence base than the preclinical literature. Maia et al. reported symptom improvement after adding 30 mg/day resveratrol to an oral contraceptive in 12 women whose pain had persisted during contraceptive treatment; however, the study was uncontrolled, and amenorrhea under continuous hormonal treatment is an important potential confounder [66]. In contrast, the randomized double-blind placebo-controlled trial by Mendes da Silva et al. enrolled 44 women and found that 40 mg/day resveratrol added to a combined oral contraceptive was not superior to placebo for pain reduction [67]. Two publications by Khodarahmian et al. reported reductions in MMP-2/MMP-9 and VEGF/TNF-α after 400 mg/day resveratrol, but these papers represent different molecular endpoints from the same randomized exploratory cohort of 34 patients rather than independent replications [68,69]. Accordingly, molecular signals in small exploratory studies should not be interpreted as established clinical efficacy. Table 3 presents the resveratrol and quercetin human studies within a single evidence hierarchy. The principal molecular pathways reported for quercetin and resveratrol in endometriosis are summarized in Figure 3.

**Table 3:** Human clinical evidence for quercetin-containing and resveratrol interventions in endometriosis.

Study/Polyphenol	Design and Population	Intervention/Exposure	Primary Endpoints	Main Findings	Major Limitations/Risk of Bias
Signorile et al. (2018) [14] Quercetin-containing formulation	Three-arm controlled study; *n* = 90, stage IV endometriosis (30/group). Placebo arm included. Randomization is reported in later evidence syntheses, but the original report gives limited detail on allocation concealment/blinding.	Quercetin-containing multicomponent nutraceutical versus partial-formulation comparator versus placebo. Quercetin 200 mg within the formulation; omega-3/6, nicotinamide, 5-MTHF, turmeric and parthenium co-administered; twice daily for 3 months. Quercetin purity not reported; no concomitant hormonal therapy reported.	VAS symptoms; serum PGE2; CA-125; lesion-related outcomes.	Reported symptom improvement and reductions in PGE2 and CA-125 in the multicomponent group.	Quercetin effect cannot be isolated; complex co-intervention; limited reporting of concealment/blinding; product-related investigator interest.
Fadin et al. (2020) [65] Quercetin-containing formulation	Open-label, uncontrolled supplementation study; *n* = 33; clinical diagnosis of endometriosis for ≥3 months; disease stage not reported.	Quercetin 200 mg/day + Curcuma longa extract 210 mg/day (95% curcuminoids) + N-acetylcysteine 150 mg/day for 2 months; no comparator. Quercetin purity and concomitant hormonal therapy not reported.	Dysmenorrhea; pelvic pain; dyspareunia; NSAID use.	Reported reductions in pain domains and NSAID use (*p* < 0.001).	No randomization, blinding or control arm; multicomponent intervention; placebo effect and natural symptom fluctuation cannot be excluded.
Maia et al. (2012) [66] Resveratrol	Non-randomized, uncontrolled pain cohort (*n* = 12) plus separate tissue-expression analysis (*n* = 42); disease stage not specified in the report.	Resveratrol 30 mg/day added to continuous combined oral contraceptive therapy for 2 months; resveratrol purity not reported.	Pelvic pain/dysmenorrhea; aromatase and COX-2 expression.	82% complete pain resolution reported in the pain cohort; lower aromatase/COX-2 expression with combined therapy.	Very small uncontrolled symptom cohort; background hormonal therapy and amenorrhea are major confounders; separate cohorts/endpoints.
Mendes da Silva et al. (2017) [67] Resveratrol	Randomized, double-blind, placebo-controlled trial; *n* = 44 women with laparoscopically diagnosed endometriosis; stage not specified in the trial summary.	Combined oral contraceptive + resveratrol 40 mg/day versus combined oral contraceptive + placebo for 42 days (2 cycles); resveratrol purity not reported.	Pain scores; CA-125; prolactin.	Resveratrol was not superior to placebo for pain reduction.	Small and short trial, but the strongest blinded placebo-controlled clinical evidence among the reviewed polyphenol studies.
Khodarahmian et al. (2019) [69] Resveratrol	Randomized exploratory placebo-controlled trial; *n* = 34 (17/group); stage III-IV endometriosis/infertility. Same cohort as the 2021 report.	Routine endometriosis protocol + resveratrol 400 mg/day versus routine protocol + placebo for 12–14 weeks; resveratrol purity not reported.	MMP-2/MMP-9 mRNA/protein; serum/endometrial-fluid measures.	Reduced MMP-2 and MMP-9 after intervention.	Small exploratory cohort; molecular surrogate endpoints; not an independent cohort from the 2021 publication.
Khodarahmian et al. (2021) [68] Resveratrol	Randomized exploratory placebo-controlled trial; *n* = 34 (17/group); stage III-IV endometriosis/infertility. Same cohort as the 2019 report.	Routine endometriosis protocol + resveratrol 400 mg/day versus routine protocol + placebo for 12–14 weeks; resveratrol purity not reported.	VEGF and TNF-α gene/protein expression in eutopic endometrium.	Reduced VEGF and TNF-α expression after intervention.	Same cohort as 2019 report; small sample; molecular rather than symptom/lesion efficacy endpoints; not an independent replication.

## 6. Challenges in Polyphenol Research for Endometriosis

One of the primary challenges is their low bioavailability, which limits their effectiveness in clinical applications. Many polyphenols are poorly absorbed in the gastrointestinal tract, undergo rapid metabolism, and are excreted before exerting their full therapeutic effects.

Encapsulation technologies, including liposomes, nanoparticles, and polymer-based systems, have been investigated as strategies to improve polyphenol stability, solubility, exposure, and tissue delivery. Liposomes are attractive because lipid composition and manufacturing parameters can be modified, but encapsulation efficiency, biodistribution, release kinetics, and organ accumulation depend on both the physicochemical properties of the polyphenol and the carrier. These approaches may improve pharmacokinetic performance, but the present evidence does not establish that advanced formulations are clinically more effective or cost-effective in endometriosis [70].

Another limitation is the lack of standardized dosing and formulations. Although many polyphenols have favorable safety profiles, high doses may produce adverse effects, including gastrointestinal symptoms and clinically relevant interactions with medicines [71]. The production of polyphenol-based nanoformulations needs the establishment of straightforward and universal methodologies, along with the advancement of rational design and synthesis tailored to specific needs to prevent the occurrence of these unwanted side effects. New approaches, such as combining chemical grafting with supramolecular self-assembly, could offer promising solutions to address these challenges, while improving the safety and performance of both the polyphenol payload and the nanocarrier [10].

For future pharmaceutical or nutraceutical development, sourcing and extraction also affect batch-to-batch composition, purity, residual solvents, and scalability. Standardized, lower-toxicity extraction and quality-control procedures may improve reproducibility while reducing the environmental burden associated with processing phenolic-rich raw materials or agro-industrial by-products [72,73].

A further consideration, and one that is often understated in reviews advocating polyphenol supplementation, is that the redox behaviour of these compounds is concentration-dependent and not uniformly protective. At the higher concentrations achievable with concentrated extracts, quercetin, resveratrol and the catechins can autoxidise in the presence of transition metals to generate semiquinone radicals, quinones and hydrogen peroxide, and can reduce Fe(III) to Fe(II) and thereby sustain rather than interrupt Fenton chemistry [11]. Given the iron-loaded peritoneal environment described in The Redox Microenvironment of Endometriotic Lesions above, this pro-oxidant potential is not a theoretical concern in endometriosis specifically, and it has not been systematically evaluated in any of the human studies reviewed here. Clinically, the best documented example of dose-dependent harm is hepatic: the European Food Safety Authority concluded that daily doses of epigallocatechin-3-gallate at or above 800 mg from concentrated green tea preparations are associated with elevations in serum transaminases in a minority of exposed individuals, and was unable to establish a safe upper level for supplemental catechins [71].

Compound-specific estrogenic activity is a central translational consideration rather than a peripheral safety issue in an estrogen-dependent disease. Resveratrol, genistein and related structures can bind estrogen receptors and display agonistic or antagonistic behavior depending on concentration, receptor subtype, metabolism, and the prevailing endogenous estrogen milieu, as reflected in Table 2 [20,31]. Consequently, an exposure that is antiproliferative in one experimental context cannot be assumed to have the same effect in another, and sub-optimal or differently metabolized exposures could theoretically produce divergent responses. Trials of phytoestrogenic polyphenols in endometriosis should therefore include explicit dose-ranging, pharmacokinetic assessment, and estrogen-responsive endpoints rather than extrapolating a generalized ‘polyphenol effect.’

Furthermore, the effects of polyphenols in human endometriosis are difficult to evaluate because the local inflammatory state cannot yet be quantified reliably with a single non-invasive marker. Pain is multifactorial and may arise from inflammatory signaling, fibrosis, adhesions, anatomical distortion, neuropathic mechanisms, or combinations of these processes; therefore, changes in pain do not directly identify an anti-inflammatory mechanism. The interpretation is further complicated when polyphenols are administered together with hormonal therapy or as multicomponent nutraceuticals. Polyphenols should therefore be investigated as candidate adjunctive interventions in trials that compare standardized formulations against appropriate controls and clearly separate background therapy from the experimental intervention.

## 7. Discussion

This review identifies recurring mechanistic themes among selected polyphenols, including modulation of redox-sensitive pathways, inflammation, angiogenesis, estrogen signaling, fibrosis, and cell survival. These themes should not be interpreted as a uniform class effect. Resveratrol and quercetin have generated the greatest clinical interest, but their prominence primarily reflects the availability of human studies, while other compounds remain confined to heterogeneous preclinical models. Differences in structure, metabolites, receptor activity, and exposure substantially limit direct comparison across compounds.

Redox biology is particularly important to this interpretation. In endometriosis, the aim cannot be reduced to global suppression of oxidative stress because ROS participate in both pathological tissue injury/inflammatory signaling and regulated stress responses or cell-death pathways. Polyphenols may alter this network through direct chemical redox reactions, Nrf2/KEAP1 signaling, mitochondrial effects, metal-dependent pro-oxidant reactions, or indirect effects on inflammation and metabolism. The net result is therefore compound-, dose-, metabolite-, cell-, and disease-context-dependent. This framework helps reconcile why apparently ‘antioxidant’ compounds may also promote ROS-associated apoptosis in experimental lesion cells and why indiscriminate antioxidant supplementation cannot be assumed to be beneficial.

The principal translational implication of the preclinical literature is that polyphenols merit further study as candidate adjunctive interventions, not that an adjunctive clinical role has already been established. Their multi-target effects provide testable hypotheses for combination with standard care, but clinical benefit and synergy cannot be inferred from pathway modulation alone. The discrepancy between large effects in controlled cell/animal systems and variable human outcomes likely reflects differences in models, dose and exposure, pharmacokinetics, lesion phenotype, concomitant therapy, and the biological heterogeneity of human endometriosis.

The human evidence base also requires explicit appraisal of study quality. Table 3 includes six reports involving quercetin-containing or resveratrol interventions, but these do not represent six independent high-quality efficacy trials. The quercetin evidence derives from multicomponent formulations: the Signorile study used a controlled three-arm design but could not isolate the effect of quercetin, whereas the Fadin study was open-label and uncontrolled [14,65]. For resveratrol, Maia et al. was uncontrolled, the two Khodarahmian publications arose from the same 34-patient cohort and focused on molecular endpoints, and only the Mendes da Silva trial was double-blind and placebo-controlled for a clinically relevant pain outcome; that trial did not show superiority over placebo [66,67,68,69]. Thus, positive human signals arise mainly from uncontrolled, multicomponent, or small exploratory studies, while the strongest pain-efficacy trial was negative.

Dose selection, pharmacokinetics, and safety require equally cautious interpretation. Many polyphenols are consumed safely in foods, but dietary exposure cannot be extrapolated automatically to long-term supplemental or pharmacological dosing. The endometriosis literature provides little long-term safety information, and high-dose preparations may introduce gastrointestinal adverse effects, drug interactions, hepatotoxicity for selected compounds, pro-oxidant behavior, or phytoestrogenic effects. Future trials should therefore include formal adverse-event monitoring, pharmacokinetic characterization, dose-ranging, and predefined safety criteria rather than assuming suitability for chronic use.

Several limitations should be considered when interpreting the current evidence. Clinical studies are small and methodologically heterogeneous, and long-term efficacy and safety data are limited. More fundamentally, the term polyphenol encompasses compounds with markedly different pharmacokinetics, metabolites, redox potentials, estrogen-receptor interactions, and effective concentrations. Experimental models and outcome measures are also heterogeneous, and many in vitro exposures are not directly comparable with systemic human exposure. These differences preclude a unified therapeutic profile for polyphenols as a class and require compound-specific evaluation from mechanistic studies through dose-ranging and clinical trials.

Endometriosis is a complex estrogen-dependent inflammatory disease in which immune dysregulation, disturbed redox homeostasis, altered steroid signaling, neuroangiogenesis, fibrosis, and lesion-specific cellular adaptations interact. The preclinical actions reported for several individual polyphenols are mechanistically relevant to this network, but they do not establish clinical efficacy in patients. Resveratrol and quercetin currently appear prominent mainly because they have reached human investigation; this should not be interpreted as evidence that they are intrinsically superior to other compounds or that either has an established adjunctive role.

However, this apparent superiority may partly reflect the disproportionate availability of clinical data rather than an intrinsic difference in biological efficacy. Other polyphenols, although extensively studied in preclinical settings, have not yet been evaluated in human populations, limiting direct comparative assessment.

Low and variable bioavailability, non-standardized preparations, uncertain metabolite activity, and limited dose-ranging and safety data remain major barriers to translation. Future research should determine whether a standardized polyphenol intervention adds clinically meaningful benefit to established care before any therapeutic role, accessibility advantage, or economic benefit is assumed.

Taken together, current evidence justifies further evaluation of polyphenols as candidate adjunctive interventions rather than supporting an established adjunctive role. Possible synergy with hormonal, analgesic, or surgical strategies remains a hypothesis that requires prospective testing with standardized preparations, appropriate comparators, clinically relevant endpoints, and adequate statistical power.

## 8. Future Directions

One of the most significant gaps in the current evidence is the lack of adequately powered, well-controlled human studies evaluating defined polyphenol interventions in endometriosis. Existing human research is limited mainly to resveratrol and quercetin-containing formulations, while other compounds remain preclinical. Future work should prioritize compound-specific dose-ranging and pharmacokinetic studies before assuming that findings can be generalized across the polyphenol family. Randomized trials should evaluate standardized formulations, clinically achievable exposure, concomitant hormonal therapy, estrogen-responsive outcomes where relevant, pain and quality-of-life endpoints, lesion-related outcomes, and long-term safety. Studies of combinations should be designed to determine whether additive or synergistic benefit exists rather than assuming such effects from mechanistic plausibility.

Another important research priority is the development of delivery systems that improve polyphenol bioavailability. Currently, polyphenol-containing nanoformulations face several limitations, including insufficient physiological stability or biodegradability, suboptimal drug encapsulation and loading efficiency, limited stimulus responsiveness, poor traceability, and a lack of active targeting capabilities. Systematic evaluations of the biosafety and in vivo behavior of polyphenol-based nanoformulations are essential. Future research should prioritize their targeting efficiency toward damaged or inflamed tissues, long-term toxicity profiles, in vivo biodegradability, renal clearance mechanisms, and interactions with biological systems.

## 9. Conclusions

Individual polyphenols can modulate processes implicated in endometriosis, including NF-κB- and MAPK-dependent inflammatory signaling, VEGF-related angiogenesis, matrix remodeling, estrogen-related signaling, apoptosis, and Nrf2-regulated redox responses. However, these compounds should not be treated as a single functional or pharmacological group. Their effects depend on structure, dose, metabolism, receptor activity, cellular context, and experimental model, and redox modulation may include antioxidant, signaling, or pro-oxidant effects. The preclinical literature therefore provides mechanistic support for further compound-specific investigation rather than proof of a class-wide therapeutic effect.

Human evidence remains preliminary and is limited to resveratrol and quercetin-containing interventions. Quercetin has not been adequately evaluated as monotherapy, and the resveratrol literature includes one double-blind placebo-controlled pain trial that was negative, alongside uncontrolled or small exploratory studies reporting clinical or molecular signals. Variable bioavailability, uncertain metabolite activity, non-standardized preparations, dose-dependent pro-oxidant or phytoestrogenic behavior, and limited long-term safety data remain unresolved. On current evidence, polyphenols are best regarded as potential adjunctive approaches whose clinical efficacy remains to be established in adequately powered, compound-specific randomized trials.

## Figures and Tables

**Table 1:** Classification, chemical structure and main sources of polyphenols; EGCG, epigallocatechin gallate.

Chemical Structure	Specific Structural Characteristics	Main Representatives	Main Sources	Reference
Flavonoids				
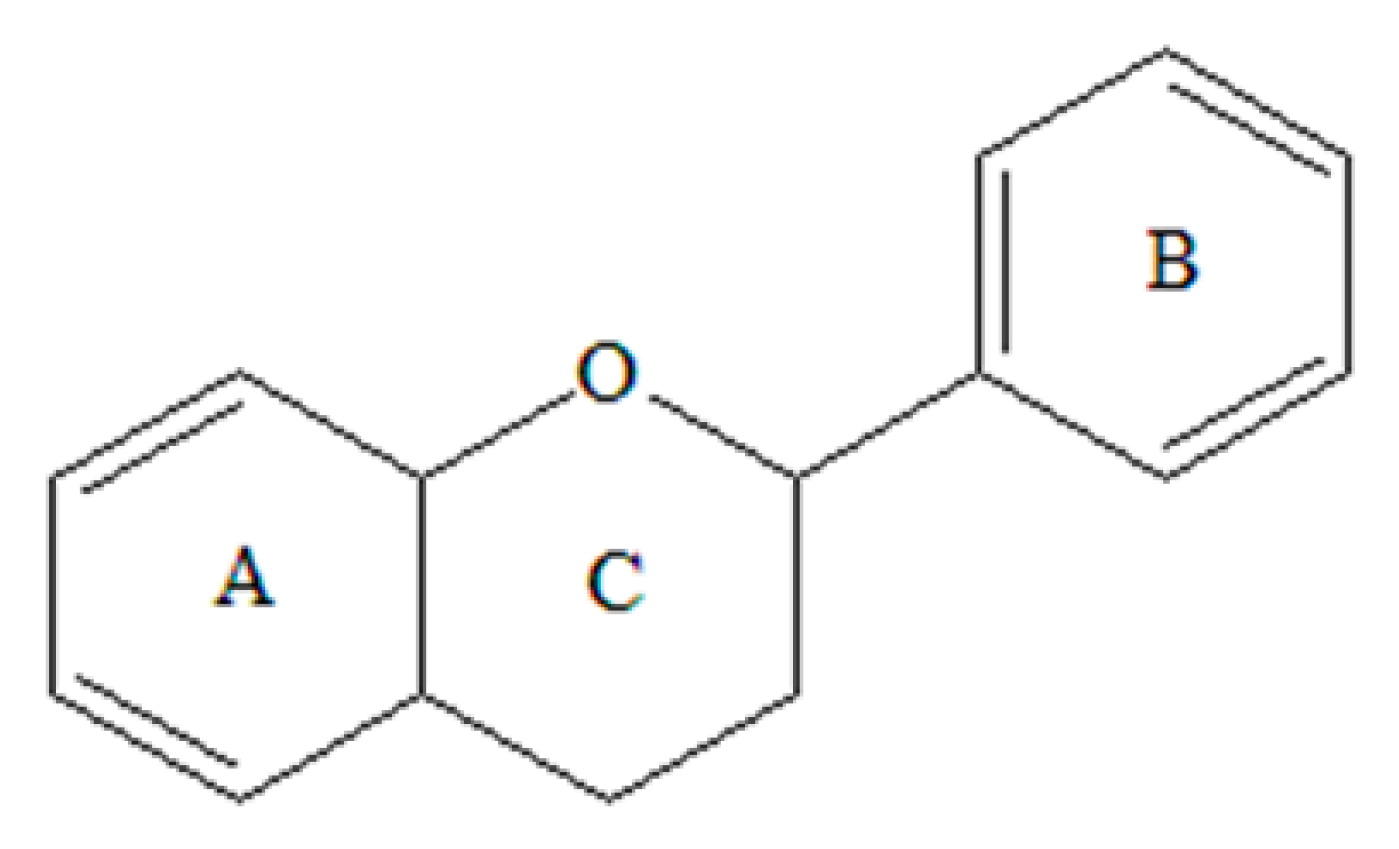	-C6–C3–C6 backbone; -Two phenolic rings (A and B) and one chromane ring (C).	-Anthocyanins: Cyanidin -Isoflavones: Genistein -Flavanones: Hesperetin -Flavanols: EGCG -Flavones: Apigenin -Flavonols: Quercetin	-Tea -Berries (e.g., blackberry, chokeberry, blueberry) -Vegetables (e.g., purple carrot, red cabbage, broccoli, onion, celery, leek) -Citrus fruits	[11,21,22,23]
Phenolic acids				
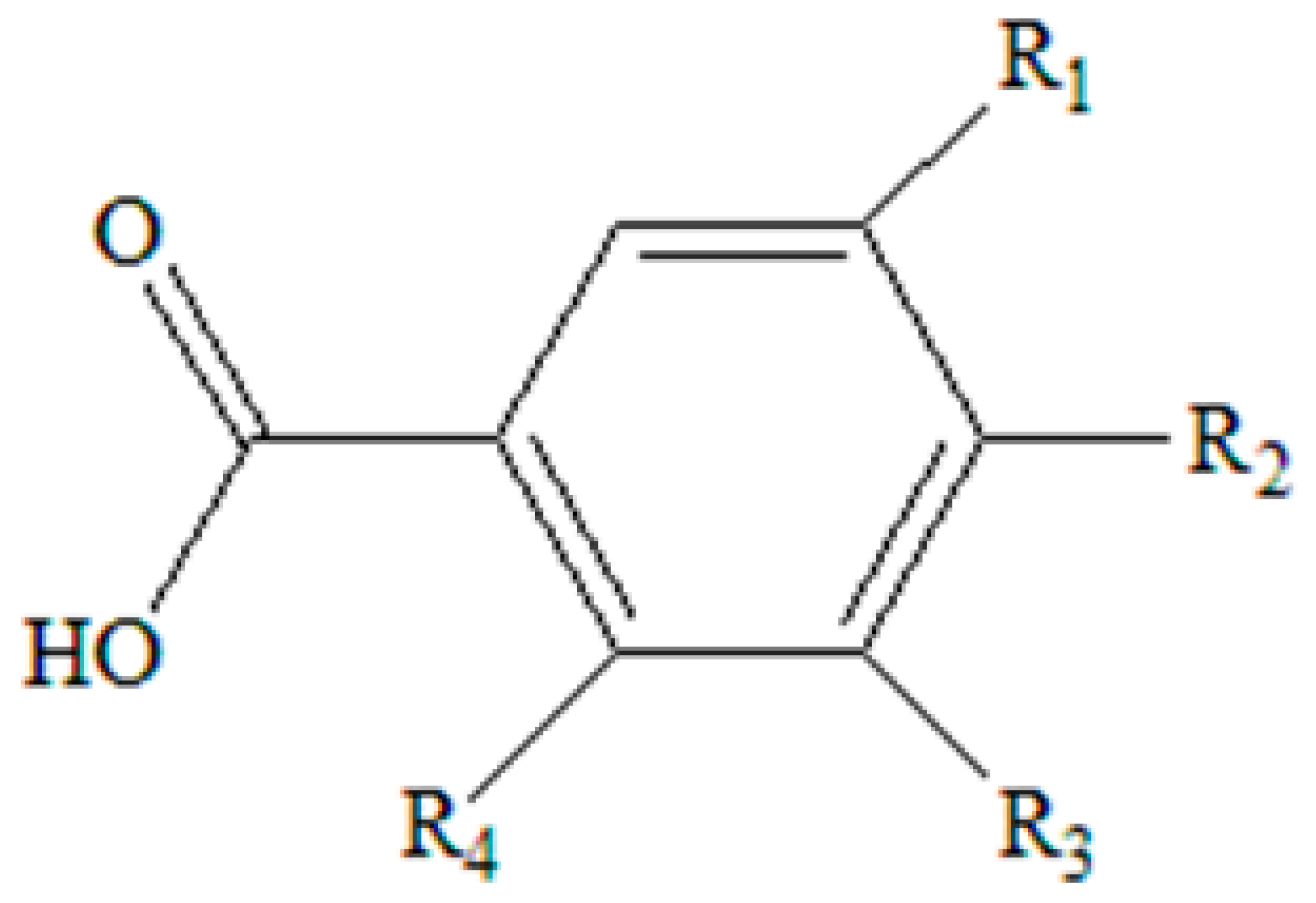	-C1–C6/C3–C6 backbones; -Up to four hydroxyl or other substituent groups.	-Hydroxybenzoic acids: Gallic acid -Hydroxycinnamic acids: Ferulic acid	-Tea, coffee -Gallnut, salvia, onion; -*Ferula asafoetida*	[11,21,23]
Lignans				
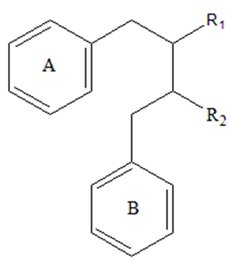	-Two phenolic rings (A and B) linked by β,β-bonds. -Can form C-C bonds between each other, forming neolignans.	-Secolariciresinol -Lariciresinol -Matairesinol -Pinoresinol	-Whole grains and seeds (e.g., barley, flax, millet, oats, sesame seeds, wheat)	[21,23,24]
Stilbenes				
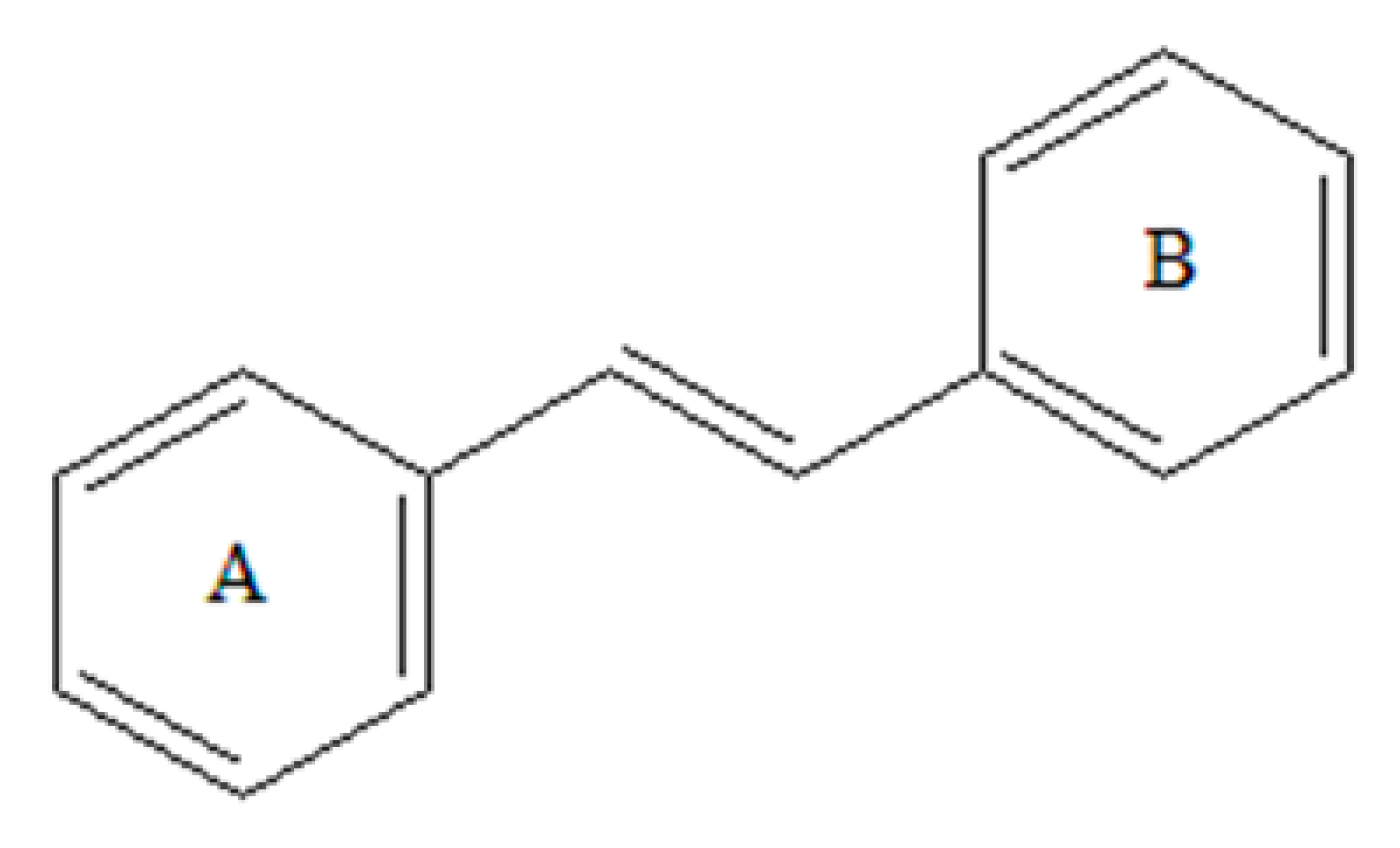	-One ethylene moiety with one phenyl group substituent on each end of the carbon–carbon double bond.	-Resveratrol -Pterostilbene	-Fruits and berries (e.g., strawberries, raspberries, mulberries, grapes) -Peanuts -Red wine	[21,23,25]

## Data Availability

No new data were created or analyzed in this study. Data sharing is not applicable to this article.

## Abbreviations

The following abbreviations are used in this manuscript:

Abbreviation	Full Term
AKT	Protein Kinase B
BAX	Bcl-2-associated X protein
CA-125	Cancer Antigen 125
CAT	Catalase
CCND1	Cyclin D1
CDK	Cyclin-dependent kinase
COX-1/2	Cyclooxygenase-1/2
CYP19A1	Cytochrome P450 Family 19 Subfamily A Member 1 (Aromatase)
DNA	Deoxyribonucleic Acid
EGCG	Epigallocatechin Gallate
EMT	Epithelial–Mesenchymal Transition
ER	Estrogen Receptor
ERK	Extracellular Signal-Regulated Kinase
FCεRI	High-affinity IgE receptor
FGF-9	Fibroblast Growth Factor 9
FSH	Follicle-Stimulating Hormone
G-CSF	Granulocyte Colony-Stimulating Factor
GPx	Glutathione Peroxidase
HGF	Hepatocyte Growth Factor
HO-1	Heme Oxygenase-1
ICAM-1/VCAM-1	Intercellular/Vascular Cell Adhesion Molecule 1
IGF-1	Insulin-like Growth Factor 1
IL	Interleukin (e.g., IL-6, IL-8, IL-1β)
IP-10	Interferon gamma-induced Protein 10
JNK	c-Jun N-terminal kinase
LH	Luteinizing Hormone
MAPK	Mitogen-Activated Protein Kinase
MCP-1	Monocyte Chemoattractant Protein-1
MKI67	Marker of Proliferation Ki-67
MMP	Matrix Metalloproteinase
NF-κB	Nuclear Factor kappa-light-chain-enhancer of activated B cells
NK	Natural Killer (cells)
NO	Nitric Oxide
NQO1	NAD(P)H Quinone Dehydrogenase 1
Nrf2	Nuclear factor erythroid 2-related factor 2
p38	p38 MAP kinase
PAK1	p21-activated kinase 1
PCNA	Proliferating Cell Nuclear Antigen
PGE2	Prostaglandin E2
PI3K	Phosphoinositide 3-kinase
PTGS2	Prostaglandin-endoperoxide synthase 2 (COX-2)
RANTES	Regulated upon Activation, Normal T Cell Expressed and Secreted
ROS	Reactive Oxygen Species
SIRT1	Sirtuin 1
SOD	Superoxide Dismutase
SRC	Steroid Receptor Coactivator
TAK1	Transforming Growth Factor Beta-Activated Kinase 1
TGF-β	Transforming Growth Factor-beta
TIMP-1	Tissue Inhibitor of Metalloproteinases-1
TNF-α	Tumor Necrosis Factor-alpha
Th1/Th2	T-helper cell types 1 and 2
VEGF	Vascular Endothelial Growth Factor
ZEB2	Zinc Finger E-Box Binding Homeobox 2
iNOS	Inducible Nitric Oxide Synthase
mTOR	Mammalian Target of Rapamycin
GPX4	Glutathione Peroxidase 4
GnRH	Gonadotropin-Releasing Hormone
4-HNE	4-Hydroxynonenal
IκBα	Inhibitor of Nuclear Factor Kappa B, Alpha
KEAP1	Kelch-like ECH-Associated Protein 1
MDA	Malondialdehyde
